# The Historical Development of the Successful Dialogues in Mental Health Model

**DOI:** 10.3390/healthcare13141696

**Published:** 2025-07-15

**Authors:** Marta Soler-Gallart, Alba Crespo-López, Garazi López de Aguileta, Mimar Ramis-Salas, Esther Oliver

**Affiliations:** 1 Department of Sociology, University of Barcelona, 08034 Barcelona, Spain; glopezdeaguileta@ub.edu (G.L.d.A.); mimarramis@ub.edu (M.R.-S.); estheroliver@ub.edu (E.O.); 2 Department of Theory and History of Education, University of Barcelona, 08035 Barcelona, Spain; albacrespo@ub.edu

**Keywords:** mental health, social impact, psychiatry, history, health

## Abstract

**Background/Objectives:** The scientific literature shows that new scientific and social priorities regarding social impacts and co-creation are leading to profound transformations in all scientific and social contexts. In the field of mental health, one dimension of this transformation is the increasing visibility of dialogic models that support the improvement of mental health. While this is very positive, it carries a risk of deformations that can lead to negative outcomes for both society and science. There is a lack of scientific research about the errors related to the new visibility of the Successful Dialogues in Mental Health (SDMH) model. The objective of this research is to clarify a certain type of error, namely when the excellent results obtained through the use of this model in particular contexts are attributed to a supposed dialogic approach of psychiatric rehabilitation, made by researchers without a degree in medicine. **Methods:** In order to clarify this error, we use a communicative methodology through a qualitative research design, oriented to unveil the main steps in the original development of the model. **Results:** The results show that the SDMH model has never presented itself as psychiatric, but instead as a social–dialogic collaboration with psychiatrists with the aim of helping individuals to overcome mental health problems. **Conclusions:** This study clarifies the purpose of the SDMH model which contributes to benefiting citizens, particularly those with mental health conditions.

## 1. Introduction

The role of dialogue in mental health can be traced back to pre-scientific eras, when mental disorders were explained through mystical beliefs and addressed using animistic practices [1]. As this historical development unfolded, mental health approaches evolved towards primarily biological and medical models [2]. Over time, the understanding of mental health has expanded to a more comprehensive view of mental health as a social problem including social, cultural, and psychological dimensions [3]. This transition was influenced by Sigmund Freud’s approach to mental health in the late 1890s, often referred to as the “talking cure,” which constituted a departure from earlier methods that primarily relied on physical treatments [4] by placing dialogue at the centre of therapeutic practices. In recent years, there has been a surge in efforts to enhance the quality of life and social inclusion of individuals with mental health disorders through a focus on dialogue.

While the use of talk as the basis of mental health treatments has been extended over time and incorporated into many models from diverse schools of thought, the role of the quality of dialogue in promoting improvements in patients’ mental health has received less attention. The current demands of society and science for social impact and co-creation have revealed the shortcomings of many existing models, which do not consider the mitigation of adverse effects and/or are based on traditional hierarchical structures between professionals and patients [5].

There is, however, a scientific framework based on the theory of dialogic society [6] that allows for analysis of the types of dialogue that evidently have positive impacts on people’s mental health. Such analyses led to the development of the “Successful Dialogues in Mental Health” (SDMH) model, which refers to the identification and creation of dialogues that have successfully contributed to improving the mental health of patients [7]. From the very beginning, SDMHs were conceived as complementary interventions that supported medical treatments, improving the health of those citizens who participated in and benefited from them, and never as a substitute for those treatments. Among the different SDMH interventions identified, Dialogic Literary Gatherings (DLGs) stand out due to their social impact. They were identified as an SDMH in 1979 by an interdisciplinary team of researchers linked to the La Verneda-Sant Martí Adult School [8], and have been implemented in more than 15.000 diverse contexts worldwide. This team, in dialogue with patients and other citizens, observed that DLGs promote friendships, support, and solidarity among participants, leading to improvements in mental health and well-being [9,10].

Following the scientific and social priorities of social impact and co-creation, more and more researchers and professionals have developed dialogic models to address the visibility of mental health, particularly the SDMH model. However, while such visibilisation is positive, if knowledge about the history of models such as the SDMH model is not clarified, there is a risk of deforming such models, leading to certain errors. These errors are detrimental for society and, in particular, for people with mental health conditions, depriving them from the mental health benefits provided by fruitful dialogues in co-creation among professionals and citizens from different backgrounds. It is therefore essential to clarify and mitigate such errors. Nonetheless, the literature reviewed so far on this topic has pointed to a lack of scientific research on errors related to the SDMH model. To contribute to filling this gap, the primary objective of this study is to clarify the errors made when the excellent results obtained with the SDMH model in particular contexts are attributed to a supposed dialogic approach of psychiatric rehabilitation, made by researchers without a degree in medicine. The secondary objectives are to unveil the main historical steps in the original development of the SDMH model, and to determine how the SDMH model has been presented since its inception.

## 2. Materials and Methods

This study provides an in-depth analysis of the historical development of the SDMH model, focusing on the clarification of the error of attributing the excellent results obtained with this model in some particular contexts to a supposed dialogic approach of psychiatric rehabilitation, as made by researchers without a degree in medicine. To this end, this qualitative research was framed within the Communicative Methodology, given its evidenced impacts on mental health in diverse fields [11,12], which involves incorporating all voices in research through egalitarian dialogue, thus enabling pioneering research conducted through co-creation and oriented to social impact—criteria that are now required in all sciences [5]. This qualitative study does not constitute a quantitative investigation, nor does it aim to offer a representative sample. This analysis is part of a sustained and rigorous process of egalitarian dialogue undertaken by the research team. Since the inception and implementation of the SDMH model, there has been an egalitarian dialogue concerning its characteristics and impacts between the researchers and citizens involved. This study includes an in-depth analysis of existing documentation and five in-depth communicative interviews with individuals involved in the co-creation of the SDMH model (no more interviews were carried out due to saturation). The review of existing documents enabled an analysis of the objective evidence available for the SDMH model. The participants added their perceptions regarding the historical development of the SDMH model.

### 2.1. The Context of the Study: La Verneda-Sant Martí Adult School

La Verneda-Sant Martí Adult School, established in 1978 in Barcelona, was created to address the literacy needs of the population [13]. From its inception, this school has been grounded in the principles of dialogic learning [14]. It was the first to be established as a Learning Community and, as such, it implements Successful Educational Actions (SEAs), based on scientific evidence demonstrating their social impact on the lives of individuals and the broader community [15]. Among the SEAs implemented, Dialogic Literary Gatherings (DLGs) can be highlighted, which were initiated in 1979. DLGs are based on the collective co-creation of meaning and knowledge through engaging in dialogue around the best universal literary works [16].

In a process of co-creation with citizens and patients, the DLGs were identified by an interdisciplinary team of researchers to be successful in improving the well-being and recovery of patients with mental health conditions [8]. While DLG participation had been recommended by health professionals to patients from that neighbourhood for many years, in 2017 the DLGs were introduced at the primary healthcare centre (PHC) in response to participants’ demand as well as the PHC’s interest in their observed impacts on mental health [9]. In addition to DLGs, other dialogic gatherings, including Dialogic Musical Gatherings (DMGs) [17], Dialogic Feminist Gatherings (DFGs) [18], and Dialogic Pedagogical Gatherings (DPGs) [19] have also been implemented, both in this school and other contexts, with successful impacts on mental health.

This adult school has been recognised, both by those involved in it and the international scientific community, for being a space based on egalitarian dialogue among all those involved, showing respect for diversity and the inclusion of all voices in participation and decision-making processes [14].

### 2.2. Data Collection

This qualitative study was conducted through two different data collection techniques: document analysis and in-depth communicative interviews. The sources included both the existing documentation on the SDMH model and individuals involved in the co-creation of the SDMH model.

### 2.3. Document Analysis

The research team conducted a search and analysis of existing documentation on the SDMH model. They agreed upon the following inclusion criteria for the documentation: (1) any type/format of document, and (2) documents that provided information on the SDMH model. The search sources were (1) Web of Science and Scopus databases, to search within the scientific literature; (2) the internet search engine Google, to search for information published in news and websites; and (3) the historical archives of La Verneda-Sant Martí Adult School, to search for paper documents such as pamphlets, letters and programmes. The search keywords were ‘Successful Dialogues in Mental Health’, ‘mental health’ AND ‘Verneda-Sant-Martí Adult School’, ‘health’ AND ‘Verneda-Sant-Martí Adult School’, ‘mental health’ AND ‘Verneda’, ‘health’ AND ‘adult school’. The final corpus of documents analysed included scientific articles, website publications and paper pamphlets regarding courses held at the Verneda-Sant Martí Adult School.

### 2.4. In-Depth Communicative Interviews

Qualitative data were gathered through in-depth communicative interviews. The participants were selected through purposive sampling, and were deliberately chosen on the basis of meeting the following inclusion criteria: (1) being familiar with the history and development of the SDMH model, and (2) not having attributed the results obtained from the SDMH model to a different approach. During these interviews, the researcher introduced the objective under analysis, and the interviewee contributed their perspective and any information they deemed relevant. All participants were informed before the interview about the aims of the study and gave their consent to participate. The participants’ profiles are presented in Table 1.

### 2.5. Data Analysis

All the information gathered was analysed by the research team in egalitarian dialogue, with the aim of identifying any data that addressed the study goal and thereby contributed to the history and clarification of the SDMH model. In this process, information obtained through document analysis was extracted and the communicative interviews were transcribed, with relevant quotations subsequently selected by consensus. The analysis was conducted both inductively and deductively, forming categories arising from the data and contrasting such categories with respect to the scientific literature, thus further informing their development. Consequently, an analytical procedure was conducted to filter the retrieved scientific evidence.

## 3. Results

### 3.1. Co-Creation as the Seed for the Development of the SDMH Model

The SDMH model was born among professionals, citizens and patients and developed from the observation of the impacts of dialogue on the people engaged in La Verneda-Sant Martí Adult School, considering the characteristics common to those dialogues that have beneficial effects on mental health, as Raquel—an educator at this school for almost 20 years—explains:

“From the outset, educators were attentive, listening to the conversations of participants of the adult school with mental health issues and the dialogues that supported them, contributing to opening these collaborations with health professionals.”.

(Raquel)

Raquel specifies that educators who worked at the school referred explicitly to mental health, and spoke about its importance and how it is addressed in the school:

“Yes, those of us who were working there did talk about mental health directly because we saw it very clearly. At that time (when I started working in the school), we were already talking about it in those terms. It’s not something we analyse and think about now, no. At that time, we were aware of what we were doing and understood the importance of it.”

(Raquel)

One initiative at this school was the creation of a health centre for women, referred to at the time as a Family Planning Centre, as Julia—an educator at La Verneda-Sant Martí Adult School since its inception and co-leader of the SDMH model—explains:

“In the same building, there was the adult school, the library, the childcare centre, and the women’s health centre. A doctor from the public health system volunteered her time in that family planning space, undertaking highly committed volunteer work.”

(Julia)

Furthermore, during La Verneda-Sant Martí Adult School’s first academic year in 1978, a series of courses were organised with eight doctors from various medical fields. Julia refers to these courses conducted while she was working at the school:

“We did so many things. I recall developing a course, although I don’t remember the title, with the doctor who volunteered at the school. At the time, I was teaching biology at the school, and we conducted a health program, so we had numerous dialogues with her and “the other women” (participants in the school) about mental health and health issues. We did this health programme for several years. That was in the early 1980s.”

(Julia)

It is in this context that, in 1982, a significant change occurred with the establishment of a mental health unit within the National Health Service in the neighbourhood, marking a revolutionary development in the field of mental health [20]. The mental health unit was responsible for three neighbourhoods in Barcelona: La Mina, La Pau, and La Verneda. La Pau and La Verneda were the areas of the Adult School, and La Mina was where Marcos—the founder of La Verneda-Sant Martí Adult School and co-founder of the SDMH model—also previously worked as a civil servant teacher. This mental health unit was created and run by psychiatrist Carlos [pseudonym], who worked there for 22 or 23 years from 1982 [21]. Carlos and La Verneda-Sant Martí Adult School were in contact and engaged in informal collaborations, as both shared the same approach to understanding mental health, albeit from very different fields (rather than ‘dialogical,’ he referred to ‘community-based,’ meaning ‘community mental health’), as Julia mentions:

“We then had a long-standing relationship with Carlos’s Psychiatry Group. He was a very famous psychiatrist. Then, the psychiatrist and mental health group, who were in Sant Martí, sent people with mental health issues to the school. It lasted for many years, and we had a really smooth relationship. We knew it because people came and told us, ‘The Carlos group recommended that I come here.’ That is why a few years ago, Carlos was the town crier for the Verneda festivities.”

(Julia)

In addition to the medical team led by this psychiatrist, other medical specialists have recommended their patients to attend La Verneda-Sant Martí Adult School because of its positive impacts on mental health, always seeking collaboration and complementary interventions. As Raquel points out:

“The school is highly regarded. I have met people who said they came to the school on the recommendation of their primary care doctor, not a mental health professional or the Carlos team. Even people from Granollers—I recall one participant who came from there because his doctor had recommended it.”.

(Raquel)

**Figure 1:**
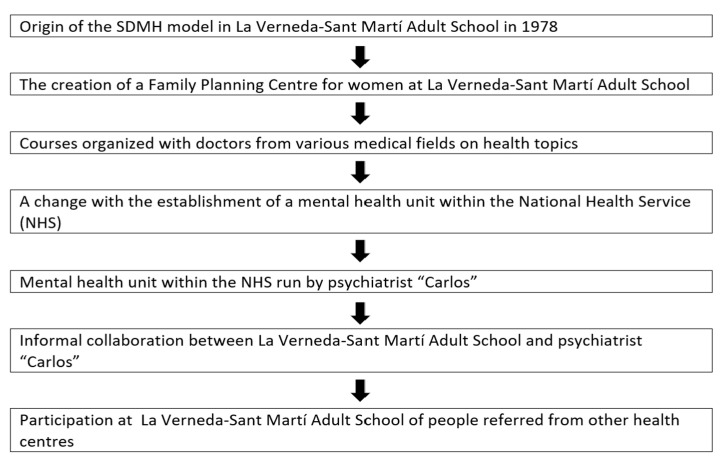
Timeline of data presented in Section 3.1.

Figure 1 shows the timeline of the data presented.

### 3.2. The Origins of SDMH Detached from Psychiatric Intervention

Mental health treatment has traditionally relied on the confinement and isolation of individuals with severe conditions—often through institutionalisation—even dating back to the 19th century. However, the practice of isolating patients has been subject to critique throughout history, as a phenomenon that cannot be attributed solely to the 1960s or any single period, with both support for and opposition to such approaches having coexisted over time [22,23]. Nonetheless, in the context of the social movements of the 1960s, there arose not only significant opposition to the confinement of those considered mentally ill—especially from a perspective understood as that of a biologist, that is, involving interventions such as medication, electroshock therapy, and so on—but also a questioning of the very notion of mental health itself and the label of mental health. Frequently, this critique stemmed from influences such as Freudo-Marxism, which posited that it is, in fact, society that is unwell. Some people have even suggested that the people that are mentally ill are in fact the ones ‘outside’ of the mental institutions, and that the others (the patients) are in fact the healthy ones [24].

Within this context of openness in the 1960s, multiple differing ideas concerning mental health converged and intermingled. In this setting, the notion of anti-psychiatry, influenced by the psychiatrist David Cooper in 1967 [25], also gained prominence, becoming an idea that rose to popularity following the May 1968 movements in circles labelled as “revolutionary.” Nonetheless, scientific evidence of the social impact of anti-psychiatry remains scarce, and those who remained committed to this notion defended their position by arguing that anti-psychiatry is ineffective because of the capitalist system. The founder of La Verneda-Sant Martí Adult School and co-leader of the SDMH explains that he had a trajectory of SDMH dialogues with professionals of mental health practice and that in his early career, in the 1960s, he was involved in a student movement alongside colleagues who initially adhered to ideas associated with anti-psychiatry, but who soon radically distanced themselves from it:

“This group was marked by a kind of ideological ferment, blending elements of anti-psychiatry and Marxist psychoanalysis. I was among them, part of that circle. However, we soon positioned ourselves in opposition to the idea of anti-psychiatry. David Cooper, closely associated with Foucault, was one of its leading proponents. In his book, *The Death of the Family*, for instance, Cooper advanced arguments that we now view as emancipatory, but which were not perceived as such at the time. Moreover, it was particularly troubling that both Cooper and Foucault supported the decriminalisation of rape and the defence of pederastia. We were unequivocally and radically opposed to these ideas.”

(Marcos)

In this context, Marcos explains that while he was living a clandestine life, opposing the Francoist dictatorship, he was connected to a group of individuals who were deeply committed to and socially aware of issues related to mental health:

“The leader of this group was Miguel [pseudonym], a highly democratic and progressive figure who was expelled from the university in 1968 as a result of a democratic movement against Franco. He later went on to work in a mental health institution in 1973. Marcos notes that, while Miguel did not establish a formal movement, he did initiate a critical reflection on mental health practice, effectively leading the broader process of collective engagement. Dialogic approach to psychiatry was therefore developed in those alternative environments of the time, and in strong collaboration with dialogic social and educational movements that focused on the improvement of the living conditions of all people.”

(Marcos)

Marcos furthermore explained the relationships between various people who would influence the approach to mental health through dialogue and the origin of the SDMH model:

“Still under the Franco regime, a clandestine meeting took place, addressing various democratic issues, including mental health. At that weekend meeting, three people met and got to know each other through me, who were Miguel [pseudonym], Jesús [pseudonym] from the Basque Country, and Isabel [pseudonym], the doctor who would later establish the Family Planning Centre for women in conjunction with the La Verneda-Sant Martí Adult School.”

(Marcos)

At the same time, in the early 1980s, a Finnish team had developed a psychiatric intervention based on a specific form of dialogic approach, which they called Open Dialogue [26]. The Open Dialogue approach is a family-oriented early intervention model for mental health problems developed in the health district of Western Lapland, where it has been implemented within the psychiatric service system [27]. At that time, alternative circles were highly interconnected throughout Europe, thus exchanging knowledge. There were connections between these models developed in Finland and the SDMH model in Spain, as Marcos explains, referring to conversations with one of his friends, Andrés [pseudonym]—a psychiatrist who was interested in the Finnish model with the dream of implementing it in the Basque Country:

“We (Marcos, Andrés and Jesús) were inseparable friends since we were eight years old. Andrés, is a psychiatrist who has been responsible for child psychiatry at the National Health Service in the entire province of Bizkaia for years. He kept in touch, he was very attentive to dialogic psychiatry in Finland, sending me publications and materials, and used to say, ‘Hey, they’re doing what you said should also be done, in psychiatry.’ So, we’ve kept in touch. And in fact, together with Andrés, we’ve always had the idea of doing it in the Basque Country.”

(Marcos)

Despite the connections, the concept of ‘dialogic psychiatry’ is attributed to the Finnish team, who were developing and practicing it [28]. The Open Dialogue approach considers people with mental health issues and their family and social network [29] as active participants, rather than as objects of treatment [30], which has demonstrated good outcomes in the treatment of first-episode psychosis [31] when working with severely disturbed psychiatric patients in crisis, among other conditions. In La Verneda-Sant Martí Adult School, psychiatric treatments were never conducted, as Julia explains:

“We did not follow up with participants to see whether or not they went to a psychiatrist; we did not do that. Some explained it, and if someone did not, we did not know and did not ask them. Holding respect for each person to explain whatever they wanted.”

(Julia)

### 3.3. Types of Dialogues That Generate Social Impact on Mental Health

The co-founder of the SDMH model explain that the SDMH model is scientifically focused on identifying the types of dialogue that improve mental health, rather than on maintaining one conception or another:

“In that context there was a fight in psychoanalysis between the most orthodox Freudians and the Lacanians, and there was quite a lot of controversy about mental health conceptions. The SDMH model within La Verneda-Sant Martí Adult School was not aimed to subscribe to one theoretical school of thought or another, but to analyse dialogues and investigate the characteristics that made them successful in improving mental health issues.”

(Marcos)

As Julia states, that is why this adult school consistently adopted the SDMH model of mental health, with the aim of identifying effective interventions. This approach led to overcoming the ‘labelling’ of a mental health condition (such as schizophrenia, eating disorder or depression), and solely considered obtaining the necessary information regarding how to provide the person with the best possible support, without disclosing their personal information to the rest of the school:

“I don’t know if they knew about specific health problems because, to me, they were all the same; they weren’t labelled (…) They did not feel they were treated as ‘poor thing, they have this’, but rather as just one more among the others.”

(Julia)

This school was founded with a dialogic approach based on the principles of dialogic learning, which ensures that interactions at the school are based on egalitarian dialogue with a deep creation of meaning, thanks to participants’ opportunity to talk about their emotions and experiences and have them valued equally [14], as well as the opportunity to acquire meaningful learning. As Raquel explains:

“The key, or what I have observed from different participants based on what they have told me, is ultimately the ‘dialogic learning’. Because when each one told me how important it was for them, they were describing almost a different principle of dialogic learning. For example, one mentioned the importance of meaning, of having something to get up for, because for her, volunteering made a lot of meaning. Or, for another, the topic of dialogue and being able to talk to other people and express what she was feeling was very important. For another, it was extremely important to be able to learn something she had never learned in her entire life, which was to read and write. For another, it was that people accepted her as she was, which is what we call equality of differences, etc. So, the fact that the school was based on dialogic learning was what also brought about this transformation in them and gave them a reason to get up every morning, because many of them said that they didn’t feel like getting up in the morning, and having a space like the school made them want to. And that saves lives.”

(Raquel)

In this regard, one of the aspects that has ensured the success of these dialogues is that they are based on truth and a real and genuine interest in people. As Raquel states:

“What is specific to this school that you would not find elsewhere is the principles of dialogic learning. Meaning, transformation… these seven principles are like a whole, the dialogic, that it is dialogic and not just talking for the sake of talking (…) here there is real dialogue, you take an interest in the person, you ask them questions, and people know that it is totally genuine because you really mean it and it is not because you are paying someone, but because it is a source of feeling, of truth, and it is free.”

(Raquel)

At this school, participants are part of a dialogic environment in which they are valued and their thoughts and feelings are taken into account, regardless of whether they want to communicate or not. This approach is ensured not only by one professional but by the whole school, as it is considered the professional’s obligation to provide an environment where participants feel valued and important. In this context, the success of dialogues in improving mental health is clearly characterised by one key feature: that the person feels deeply valued [14]. To implement this approach, the adult school established the idea of ‘specific cases,’ referring to any individual who may require additional assistance due to a wide diversity of conditions, such as suffering a physical disability or a mental health issue, among others. The person responsible for specific cases was the only one who kept the files on individuals included as ‘specific cases,’ and would act as an advisor if anything needed to be taken into account in the school to ensure the best conditions for that person. As Raquel explains:

“In specific cases, there were girls who had been victims of trafficking, people with neurodiversities, people with autism, schizophrenia, etc. Sometimes we coordinated with other entities and sometimes we didn’t. In other words, you could detect a specific case and maybe that person had schizophrenia, but you didn’t call it schizophrenia, first because you didn’t know, and second because you didn’t have to know. At the school, we have always tried to treat each person in the best way possible for them; we didn’t need a label (…) And how do you find out what they need? Through dialogue. Taking each person into account, encouraging those who speak less to speak more, ensuring they can speak, that they have their space, etc. And all of that is part of working on the whole issue of mental health.”

(Raquel)

The success of the SDMH model in ensuring dialogues in which people with mental health issues feel included and valued is reflected in the words of Julia, who shares that one participant confessed that this was the reason she participated in the school, even though she lived far away:

“At one point, we had more than 100 cases in specific cases. Then one day I asked one of them, ‘But why do you come here from so far away?’ And it really stuck with me, because she said, ‘Because here they treat us like people.’ That really stuck with me.”

(Julia)

The SDMH model in La Verneda-Sant Martí Adult School has been evidenced to promote interactions that improve the well-being of participants by fostering feelings of support and friendship [7]. As Julia further explains:

“In class, mental health was always taken into account through dialogue, mutual respect, and also, I believe, through the interrelationships between friends, such as if one was feeling unwell, another would call her, etc. We didn’t call it a health programme, but it was already happening. It was happening through attendance, relationships, dialogue, providing a much better life, having friends, a good atmosphere, parties, etc. These things also improved the problem of loneliness.”

(Julia)

Raquel highlights the case of a participant who suffered Alzheimer’s disease, for whom this adult school provided successful dialogues within diverse classes and spaces:

“A lady who had Alzheimer’s and didn’t have a place in a day centre came to the new readers’ class and other classes, and the school adapted, not only in terms of dialoguing and creating meaning, making friends and discussing topics, but also in terms of welcoming everyone, all her needs.”

(Raquel)

Dialogues with these characteristics have always been promoted in all areas and in the different activities of the school, although the SEAs of the DG stand out, as Raquel points out:

“My experience is that it happens in any space (at school), because ultimately, the school is imbued with dialogic learning in all areas. (…) are activities that bring them to life because they are imbued with dialogic learning. Also in dialogic gatherings, of course, I think that’s where it happens most, but in literacy classes I have also found people who have experienced this process of transformation (…) this space of freedom.”

(Raquel)

### 3.4. Dialogic Literary Gatherings (DLGs) as a Successful Action in the SDMH Model

In a DLG, participants attend having read the agreed-upon section of the book and highlight a sentence or paragraph and their reflection, ideas or feelings on it [15,16]. During DLGs, a facilitator opens the floor for all participants to share the words they want to, equally respecting and validating all opinions for its arguments and not deferring to status or power relationships. The facilitator always favours those who have participated less and ensures that the interactions among participants are based on egalitarian dialogue and that all voices are included, which allows for the co-creation of interpretations of the text and participants’ feelings and lives. The participants sometimes had low self-esteem (for some of them, in addition to different mental health problems). This low self-esteem was influenced by the way that other people—and even their own families—treated them, which sometimes made them feel very out of place in society [13]. The DLG embodied a type of dialogue that was extraordinarily successful, as Marcos explains below:

“In the very first DLG, there were already two people with serious mental health problems. And the very fact that they were listened to, even by people with university degrees, in this case professionals, because the participants did not hold any academic degrees, the fact that they were taken seriously and listened to what they had to say, was a point of self-esteem that helped them. We never suggested that this was going to solve their mental health problems. In other words, it was a valuable aid to them, but it needed to be supplemented by professional psychiatric care. DLG was never intended as an alternative to psychiatry or as an opposition to mental health institutions. On the contrary, it was always intended as complementary and, moreover, in dialogue with those institutions. In fact, we never suggested that ‘medical intervention’ in the sense of medication or other interventions should be stopped.”

(Marcos)

These DLGs had such an impact on the mental health of participants that some of them even encouraged the transfer of this action to a primary healthcare centre for the first time [9], as Julia stated:

“Regarding the dialogic gatherings, during a school session, a woman explained in front of everyone, and there were many of us, how badly she had been, and how she participated in the dialogic gatherings, and how she improved. She was one of the pioneers who later brought dialogic gatherings to the healthcare centre.”

(Julia)

In this regard, Erin—the coordinator of the Dialogic Minds project [32], an Erasmus+ project with the main objective of fostering the social inclusion of adult mental health users with the implementation of DLGs—explains that DLGs were implemented in diverse contexts in four different countries for adults with different health issues:

“As part of this project, DLG were implemented in health centres in four different countries: a residence for people with mental health problems in Greece, a centre for people with mental health problems in Romania, some of them with psychotic disorders, a primary care centre in Spain, and an NGO working with adults with mental health problems in the Netherlands. People from these countries were trained and then moderated DLG in these institutions. An online course was also created, which is still open and continues to train more people in more contexts to implement DLG.”

(Erin)

The most successful dialogues began to be systematised and different ones were identified. However, dialogic gatherings, including DLGs, DMGs, DFGs and other types, were identified as the most effective ones. The DLGs among participants with serious mental health issues would begin with Kafka’s book, *The Metamorphosis*. The choice of this book with patients of severe mental health issues has been a constant fact, as it continues to be used in DLGs for psychotic patients (including some schizophrenics), as dialogues based on Kafka’s *The Metamorphosis* have been very successful in diverse contexts. This was also the case for the project that Erin was coordinating.

Erin adds some of the books that have been considered in these DLGs in different contexts, including Kafka’s *The Metamorphosis*:

“In these DLG with mental health patients, we have read books by Saramago, Federico Garcia Lorca, and we have dialogued Kafka’s *The Metamorphosis*. *Romeo and Juliet*, among others, has also been read.”

(Erin)

The existing scientific literature shows that the need of some DLG participants to take psychotropic drugs has been reduced [9]. Nonetheless, it has never been the aim of a DLG—nor of the SDMH model—to replace any medical treatment. Julia clarifies that it was the participants who used expressions like: “I feel so good now, I don’t take pills anymore, look at me.” The SDMH aims to include dialogic activities in a complementary manner to other treatments. It was due to the observed positive impact on mental health that word quickly spread among mental health professionals and institutions. They believed that the Adult School of La Verneda was a worthwhile endeavour, and subsequently recommended that a large number of patients attended the school:

“There were two issues here: one was that they [psychiatrists and doctors] saw that it was beneficial and helped their mental health patients with their medical treatment. Also, in many cases, when the therapy they were receiving was private, it was because they were people who did not have the resources to pay for it, so the aim was to reduce the cost as much as possible. One way therapists could reduce the cost for these individuals was to complement their work with ours, which was provided free of charge to the patients. We did this for five years, from 1978 to 1982.”

(Marcos)

Erin also highlighted the mental health impacts of the DLG identified in this project:

“On the final conference of this project, when we shared the mental health results of these DLG, some participants shared the positive impacts these DLG had had on themselves, highlighting the creation of friendships and support networks, increased self-esteem, increased respect and solidarity, and increased autonomy.”

(Erin)

Furthermore, Erin adds the positive assessment of these LGTs by health professionals and the possibility of training other professionals to implement them:

“There was also a health centre director, someone who was working at the public health agency and her boss, and they explained the impacts they had seen on health and that they believed this should be expanded. They appreciated that we provided scientific evidence and also the voices of participants on the impact that DLG has on them. In fact, we had meetings because they wanted to provide training in different health centres so that DLG could be implemented and psychologists or social workers could be trained to do so.”

(Erin)

### 3.5. Clarifying Errors

The educator at La Verneda-Sant Martí adult school, Raquel, explains the case of two academics (pseudonyms Montse and Nuria) without a degree in medicine, who attributed the excellent results obtained from SDMH model in particular contexts to a supposed dialogic approach of psychiatric rehabilitation:

“Montse and Nuria were trained in the SDMH model by its co-founder Marcos and implemented it very well when following exactly the scientific bases of the model, always with the guidance of the founder. Later, both wrote that they had created a dialogic approach to ‘psychiatric rehabilitation’, attributing this name to the excellent results obtained by the SDMH model, which never presented itself as psychiatric. To practice psychiatry, it is compulsory to first hold a university degree in medicine, and neither the founders of SDMH nor those two academics hold a degree in medicine. This model [SDMH] conducts a social dialogic collaboration with psychiatry for the overcoming of mental health issues, but only psychiatry decides the treatments, and only psychiatrists do psychiatry.”

(Raquel)

Another collaboration of La Verneda-Sant Martí adult school, Olivia—who currently moderates a DG at this adult school—provided more evidence for that error, regarding the events that occurred when Montse moved to work in another city and university and Marcos proposed that she implemented the SDMH model there. Organising a meeting between Montse and Andrés, the former mentioned very important psychiatrist who was a close friend of him, who was very interested in the dialogic approach of psychiatry practiced by the Finnish team and the SDMH model practiced by Marcos. Olivia added very precise information:

“This meeting was held in a famous Café on 19th June 2015 at 17.30 h. Montse said that she was delighted and expressed to what extent Andres appreciated Marcos. (…). Later in 2016, on the 3rd of November, Marcos and Montse agreed to submit a project on mental health with Andres to the official call for research proposals, focused on the implementation of DLGs. On the 14th of July 2017, they commented that the proposal was not considered for evaluation due to an involuntary formal mistake not related to its content. Montse clarified that her head of the research group was worrying a lot for this mistake.”

(Olivia)

Olivia provides further insight into the progression of how these researchers became familiar with and applied the SDMH model:

“Marcos and Montse planned to do a research on mental health in collaboration with the work and contract of one doctoral student who would like to take this focus on a dissertation. The first candidate for doing this work had another professional plan. Then a clinical psychology student [Nuria] wanted to apply for a predoctoral contract and Marcos proposed to Montse to tell Nuria whether she would like to do it. Nuria accepted enthusiastically and seriously followed a profound and long training on the scientific bases of the SDMH model. Later, she was in charge of the training of professionals of an excellent institution for mental health patients. The institution published the details of this training, all of them based on the concepts and practices created by the founders of the SDMH. Then, the DLG were followed by 25 patients reading and dialoguing about the book *The Metamorphosis* by Kafka. After the difficult acceptance of her research group first (including Nuria herself), and the professionals later, to begin with this book, when they saw the positive results, all of them were extraordinarily satisfied.”

(Olivia)

Julia—one of the co-founders of the SDMH model—specifies that the approach to mental health from La Verneda-Sant Martí Adult School was never intended to be a form of dialogic psychiatry, clarifying that error:

“From the adult school, it was understood that any intervention related to mental health attempting to present it as from psychiatry would have been an intrusion and completely reckless. Professionals and participants in La Verneda-Sant Martí School never accepted it being called dialogic psychiatry.”

(Julia)

As Marcos explains, the approach of the La Verneda-Sant Martí Adult School was a dialogic model of mental health focused on the dialogues that were evidenced to have an impact on the mental health of participants, rather than referring to philosophies, ideologies, or specific concepts:

“We always approached it as a dialogic model of mental health. What was the focus of the dialogic model? Because there have been many dialogues in mental health. The goal was to ensure that the dialogues established were not predetermined by philosophies, ideologies, or specific concepts. Instead, they were established based on scientific criteria that we later came to refer to as social impact. In other words, those types of dialogues that were shown to best overcome mental health problems.”

(Marcos)

Finally, the founder of this adult school further clarifies that the error of referring to these actions encompassed in the SDMH model as ‘psychiatric’ is a mistake, and has not been made by anyone who approaches the SDMH model from the context of the La Verneda-Sant Martí adult school:

“They have made the mistake of claiming to have created a model of dialogic psychiatry but this is not accurate. This is something they have said, and from our perspective, it is not the case. Dialogic psychiatry is practised by authorized professionals of psychiatry, our collaboration with them is only providing dialogues which are very successful in mental health, like the DLG and others.”

(Marcos)

## 4. Discussion

The present study provides new scientific evidence by illustrating the historical development of the SDMH model, specifically clarifying the error of attributing the excellent results obtained with this model in particular contexts to a supposed dialogic approach of psychiatric rehabilitation, as made by researchers without a degree in medicine.

One of the main findings of this study is that the origin and development of the SDMH model has been fostered through co-creation and a clear focus on improving mental health. The transformation of the conception and treatment of mental health towards increasingly dialogic approaches is in line with modern dialogic society [6], in which citizens have increasingly advocated for their right to participate in scientific processes and benefit from its advances, as stated within the Declaration of Human Rights [33]. In recent years, the European Commission has established two criteria as requirements for the projects it funds: to be co-created and aimed at achieving social impact [5]. The findings highlight that a co-founder of the concepts of co-creation and social impact also founded an adult school in Barcelona. This adult school has been recognised by citizens and also by the international scientific community, due to its impact in terms of improving the mental health of those involved in it through the different actions framed in the SDMH model [7,8,14]. Since its inception, this adult school has implemented actions to address mental health, including the diversity of voices in the community. It provides spaces for dialogue between doctors and other health professionals, educators and participants. It has created a pioneering family planning centre to address women’s well-being and identify whether they were suffering from violence, at a time when this was largely silenced [34]. It also offered courses on different areas of health to the citizens, which constituted a pioneering development within its historical context. In the SDMH model, the involvement of participants as the primary decision-makers regarding their own health was encouraged at this adult school. This conception has also been promoted by researchers on health literacy at the Harvard School of Public Health, highlighting Rima Rudd—with whom the co-founder of SDMH was in contact during the subsequent periods [35]. The impact of this school on mental health was even disseminated among some medical and psychiatric teams, who were interested in its approach as a complement to the medical treatments required by each patient; as a result, DLGs (as part of the SDMH model) began to be implemented in primary healthcare centres and other healthcare institutions [9,10,32].

As the founding vision of this school was to transform society, improving the mental health of its participants was a key objective. To that end, the SDMH model has always been aimed at analysing those dialogues with demonstrated ability to improve the mental health of participants. Through the SDMH model, the characteristics of these successful dialogues have been collected and analysed, allowing dialogue to be understood as a form of communication aimed at helping other people. These egalitarian dialogues are based on a genuine interest in the other person, through which deep meaning is co-created. These dialogues include and value the diversity of voices, ensuring that all different people feel heard, recognised and valued. They foster supportive and friendly interactions, ensuring freedom and consent for all those involved. The SDMH model addresses people’s mental health without focusing on the label of their mental issue.

In addition to La Verneda-Sant Martí Adult School, further initiatives promoted by the school have provided additional evidence for the success of the SDMH model in diverse contexts. The Erasmus Project Dialogic Minds: Transferring Dialogic Literary Gatherings to mental health across Europe (2020-1-ES01-KA204-083054) has gathered evidence on the impact and benefits of DLGs on mental health users in Romania, Greece, Netherlands and Spain [32]. Moreover, the SDMH model has been implemented in a primary healthcare centre through DLGs, demonstrating improvements in mental health by promoting friendships, support, and solidarity both among the participants themselves and within their families and communities [9]. The SDMH model has also been implemented in a specialist psychiatric hospital, where the adult education school La Verneda-Sant Martí has promoted the development of DLGs for psychotic patients [10].

This research provides evidence that the SDMH model has allowed for improved mental health from a social approach focusing on dialogues, and has never presented itself as psychiatric; instead, it serves as a social and dialogic collaboration with psychiatrists to help in overcoming mental health issues. The SDMH model is considered an approach that co-creates dialogues that have been scientifically shown to have an impact on mental health. Its intention is not to address issues that other models from other fields—such as medicine or psychiatry—deal with but, rather, to complement them. Other psychiatric models address mental health issues that the SDMH model does not consider, such as the Open Dialogue approach, which originated in Finland, that addresses mental health from a psychiatric perspective [26,27,28,29,30,31]. Although the SDMH model and the Open Dialogue approach coincide in time and in their ultimate goal of improving people’s mental health, they are proposals from different areas, with the Finnish model being the pioneering approach to address mental health from a dialogic psychiatric perspective. Thus, the main difference between the SDMH model and Open Dialogue approach is that the former is a mental health model designed to provide dialogues that are very successful in improving mental health, whereas the latter is a psychiatric approach practised by authorised psychiatrists [10,31].

The limitations of the SDMH model that have been identified are linked the fact that it is a model intended to improve mental health only as a social and dialogic model in collaboration with other medical practices, rather than a model of psychiatric treatment. A key risk associated with the SDMH model lies in the potential for professional intrusiveness by individuals who make the mistake of attributing the excellent results obtained with this model in particular contexts to a supposed dialogic approach of psychiatric rehabilitation, a mistake often made by researchers without a degree in medicine. The challenges associated with the SDMH model lie in ensuring that it is understood and implemented for what it is, rather than being misapplied or misconstrued as serving a different purpose. Therefore, this model should be considered an aid to other actions, and not an exclusive treatment on which one should rely solely. This is a potential barrier to implementing the model in different contexts or countries. This study therefore addresses the need to ethically implement mental health models in order to ensure their impact on improving both individuals’ health and overall quality of life, as the distortion of such models may undermine this objective. The effectiveness of the SDMH model is measured through the testimonies of individuals who participate in it, as well as clinical reports by professionals in the fields of psychology, medicine, and psychiatry. Upon mitigating these risks, the SDMH model has been scientifically shown to yield improvements in mental health across the diverse contexts in which it has been implemented, and does not worsen it [9,10,32].

## 5. Conclusions

Regarding the objective of this study, evidence was presented to clarify the error made when the excellent results obtained with the SDMH model in particular contexts are attributed to the supposed dialogic approach of psychiatric rehabilitation, an error made by researchers without a degree in medicine. This study underscores the importance of clarifying the historical development of the SDMH model in order to continue benefiting citizens, particularly those with mental health conditions. Not clarifying errors such as that mentioned above may harm both patients and citizens overall. At this moment in time, in which co-creation and social impact are demanded in both social and scientific contexts, these types of historical errors must increasingly be clarified, in order to continue improving everyone’s lives.

The evidence presented in this study serves to clarify the true purpose of the SDMH model, which has shown excellent outcomes in specific contexts. This understanding supports its implementation in line with its core principles, facilitating the replication of the observed results. Recognising the model as a form of social dialogic collaboration with psychiatrists that helps to address mental health challenges may encourage its continued application across an expanding range of settings and populations. When applied with a solid grasp of its foundational principles, it can be employed as intended, that is, to identify and create dialogues that have successfully contributed to improving mental health. It complements rather than replaces psychiatric treatment, thus fostering more comprehensive and collaborative care. Such clarification can enhance the effectiveness of professionals and mental health services. Furthermore, identifying the model’s key historical stages offers valuable insights into its conceptual foundations and contributes to its proper application. In addition, the SDMH model may carry political implications, as it is a scientifically validated approach that has improved patients’ mental health without generating economic or cultural burdens, is compatible with other approaches, and has consistently been associated with positive mental health outcomes in diverse contexts. Future studies may address further aspects of the impacts of the SDMH model and the characteristics that make it transferable to other contexts.

## Figures and Tables

**Table 1:** Participant characteristics.

Pseudonym	Gender	Age Range	Profile
Julia	Woman	70–75	Educator at La Verneda-Sant Martí Adult School since 1979 and co-founder of ‘Successful Dialogues in Mental Health’ model.
Marcos	Man	70–75	Founder of La Verneda-Sant Martí Adult School and co-founder of ‘Successful Dialogues in Mental Health’ model.
Raquel	Woman	40–45	Educator at La Verneda-Sant Martí Adult School since January 2006.
Olivia	Woman	25–30	Educator at La Verneda-Sant Martí Adult School since 2021.
Erin	Woman	30–35	Coordinator of the Erasmus Project Dialogic Minds.

## Data Availability

The data presented in this study are available on request from the corresponding author due to confidentiality-related restrictions.
